# μ-Bis(diphenyl­phosphan­yl)borato-κ^2^ *P*:*P*′-bis­[dicarbon­yl(η^5^-cyclo­penta­dien­yl)iron(II)] tetrachloridoferrate(III) chloro­form solvate

**DOI:** 10.1107/S1600536808014931

**Published:** 2008-05-21

**Authors:** Franz Dornhaus, Hans-Wolfram Lerner, Michael Bolte

**Affiliations:** a Institut für Anorganische Chemie, J. W. Goethe-Universität Frankfurt, Max-von-Laue-Strasse 7, 60438 Frankfurt/Main, Germany

## Abstract

The title compound, [Fe_2_(C_5_H_5_)_2_(C_24_H_22_BP_2_)(CO)_4_][FeCl_4_]·CHCl_3_, is an oxidation product of CpFe(CO)_2_PPh_2_BH_3_. One pair of phenyl rings attached to the two different P atoms are almost parallel, as are the other pair [dihedral angles = 8.7 (5) and 8.9 (5)°]. The planes of the two cyclo­penta­dienyl rings are inclined by 26.8 (7)° with respect to each other. The carbonyl groups at each Fe atom are almost perpendicular [C—Fe—C = 92.6 (6) and 94.3 (5)°].

## Related literature

For related literature, see: Kückmann *et al.* (2007 ▶).
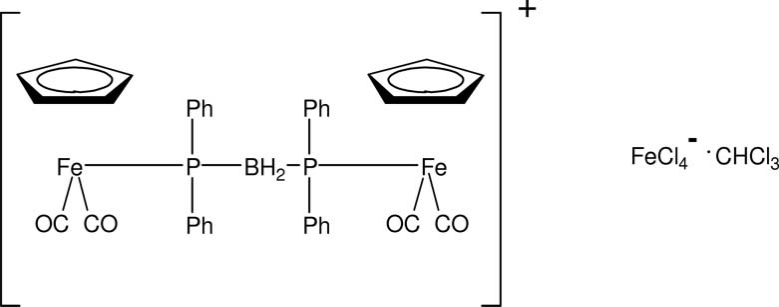


## Experimental

### 

#### Crystal data

[Fe_2_(C_5_H_5_)_2_(C_24_H_22_BP_2_)(CO)_4_][FeCl_4_]·CHCl_3_
*M* *_r_* = 1054.10
Monoclinic, 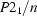

*a* = 16.0627 (17) Å
*b* = 17.8223 (17) Å
*c* = 17.205 (2) Å
β = 113.296 (8)°
*V* = 4523.8 (9) Å^3^
*Z* = 4
Mo *K*α radiation
μ = 1.47 mm^−1^
*T* = 173 (2) K
0.11 × 0.07 × 0.02 mm

#### Data collection

Stoe IPDSII two-circle diffractometer
Absorption correction: multi-scan (*MULABS*; Spek, 2003 ▶; Blessing, 1995 ▶) *T*_min_ = 0.855, *T*_max_ = 0.971
44600 measured reflections
8506 independent reflections
3324 reflections with *I* > 2σ(*I*)
*R*_int_ = 0.088

#### Refinement

*R*[*F* ^2^ > 2σ(*F* ^2^)] = 0.111
*wR*(*F* ^2^) = 0.176
*S* = 0.99
8506 reflections
505 parameters
H-atom parameters constrained
Δρ_max_ = 0.80 e Å^−3^
Δρ_min_ = −0.60 e Å^−3^

### 

Data collection: *X-AREA* (Stoe & Cie, 2001 ▶); cell refinement: *X-AREA*; data reduction: *X-AREA*; program(s) used to solve structure: *SHELXS97* (Sheldrick, 2008 ▶); program(s) used to refine structure: *SHELXL97* (Sheldrick, 2008 ▶); molecular graphics: *XP* in *SHELXTL-Plus* (Sheldrick, 2008 ▶); software used to prepare material for publication: *SHELXL97* and *PLATON* (Spek, 2003 ▶).

## Supplementary Material

Crystal structure: contains datablocks I, global. DOI: 10.1107/S1600536808014931/bq2081sup1.cif

Structure factors: contains datablocks I. DOI: 10.1107/S1600536808014931/bq2081Isup2.hkl

Additional supplementary materials:  crystallographic information; 3D view; checkCIF report

## Figures and Tables

**Fig. 1.**
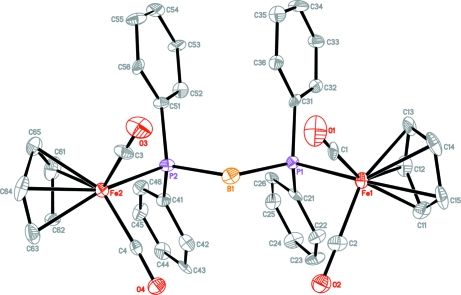
Perspective view of the title compound with the atom numbering scheme; displacement ellipsoids are at the 50% probability level; H atoms are omitted for clarity. The FeCl4 anion and the CHCl3 solvent are omitted for clarity.

## supplementary crystallographic information

### Comment

Very recently we have described the synthesis of CpFe(CO)_2_PPh_2_BH_3_ (Kückmann *et al.*, 2007). We report here air oxidation of CpFe(CO)_2_PPh_2_BH_3_ in chloroform and the result of the X-ray crystal structure analysis of the obtained oxidation product, [(CpFe(CO)_2_PPh_2_)_2_BH_2_][FeCl_4_].

The title compound is an oxidation product of CpFe(CO)_2_PPh_2_BH_3_. It is composed of cations, anions and neutral chloroform molecules. Pairs of phenyl rings attached to each of the two P atoms are almost coparallel [dihedral angles 8.7 (5)° and 8.9 (5)°]. The planes of the two cp rings are inclined by 26.8 (7)° with respect to each other. The carbonyl groups at each Fe atom are almost mutually perpendicular [C—Fe—C angle 92.6 (6)° and 94.3 (5)°].

### Experimental

In an NMR tube a solution of CpFe(CO)_2_PPh_2_BH_3_ (Kückmann *et al.*, 2007) (0.2 mmol) in 0.6 ml CDCl_3_ was exposed to air for several days at room temperature. Single crystals of the title compound were grown from this solution at room temperature.

### Refinement

H atoms were refined with fixed individual displacement parameters [U(H) = 1.2 *U*_eq_(C,B)] using a riding model with B—H = 0.99 Å, C_aromatic_—H = 0.95Å and Cl_3_C—H = 1.0 Å.

### Crystal data

**Table:**

[Fe_2_(C_5_H_5_)_2_(C_24_H_22_BP_2_)(CO)_4_][FeCl_4_]·CHCl_3_	*F*_000_ = 2124
*M**_r_* = 1054.10	*D*_x_ = 1.548 Mg m^−^^3^
Monoclinic, *P*2_1_/*n*	Mo *K*α radiation λ = 0.71073 Å
Hall symbol: -P 2yn	Cell parameters from 6332 reflections
*a* = 16.0627 (17) Å	θ = 3.6–24.5º
*b* = 17.8223 (17) Å	µ = 1.47 mm^−^^1^
*c* = 17.205 (2) Å	*T* = 173 (2) K
β = 113.296 (8)º	Plate, brown
*V* = 4523.8 (9) Å^3^	0.11 × 0.07 × 0.02 mm
*Z* = 4	

### Data collection

**Table:**

Stoe IPDSII two-circle diffractometer	8506 independent reflections
Radiation source: fine-focus sealed tube	3324 reflections with *I* > 2σ(*I*)
Monochromator: graphite	*R*_int_ = 0.089
*T* = 173(2) K	θ_max_ = 25.8º
ω scans	θ_min_ = 3.6º
Absorption correction: multi-scan(MULABS; Spek, 2003; Blessing, 1995)	*h* = −19→19
*T*_min_ = 0.855, *T*_max_ = 0.971	*k* = −21→21
44600 measured reflections	*l* = −20→18

### Refinement

**Table:**

Refinement on *F*^2^	Secondary atom site location: difference Fourier map
Least-squares matrix: full	Hydrogen site location: inferred from neighbouring sites
*R*[*F*^2^ > 2σ(*F*^2^)] = 0.111	H-atom parameters constrained
*wR*(*F*^2^) = 0.176	*w* = 1/[σ^2^(*F*_o_^2^) + (0.0133*P*)^2^] where *P* = (*F*_o_^2^ + 2*F*_c_^2^)/3
*S* = 0.99	(Δ/σ)_max_ < 0.001
8506 reflections	Δρ_max_ = 0.80 e Å^−^^3^
505 parameters	Δρ_min_ = −0.60 e Å^−^^3^
Primary atom site location: structure-invariant direct methods	Extinction correction: none

### Special details

**Table:**

Geometry. All e.s.d.'s (except the e.s.d. in the dihedral angle between two l.s. planes) are estimated using the full covariance matrix. The cell e.s.d.'s are taken into account individually in the estimation of e.s.d.'s in distances, angles and torsion angles; correlations between e.s.d.'s in cell parameters are only used when they are defined by crystal symmetry. An approximate (isotropic) treatment of cell e.s.d.'s is used for estimating e.s.d.'s involving l.s. planes.
Refinement. Refinement of *F*^2^ against ALL reflections. The weighted *R*-factor *wR* and goodness of fit *S* are based on *F*^2^, conventional *R*-factors *R* are based on *F*, with *F* set to zero for negative *F*^2^. The threshold expression of *F*^2^ > σ(*F*^2^) is used only for calculating *R*-factors(gt) *etc*. and is not relevant to the choice of reflections for refinement. *R*-factors based on *F*^2^ are statistically about twice as large as those based on *F*, and *R*- factors based on ALL data will be even larger.

### Fractional atomic coordinates and isotropic or equivalent isotropic displacement parameters (Å^2^)

**Table:**

	*x*	*y*	*z*	*U*_iso_*/*U*_eq_	
B1	0.7751 (9)	0.2979 (10)	0.8313 (8)	0.031 (3)	
H1A	0.8093	0.3431	0.8600	0.037*	
H1B	0.8119	0.2541	0.8609	0.037*	
Fe1	0.70461 (11)	0.29478 (10)	0.99844 (10)	0.0235 (4)	
Fe2	0.92129 (11)	0.30470 (10)	0.72844 (10)	0.0228 (4)	
P1	0.66442 (19)	0.29568 (18)	0.85413 (17)	0.0197 (6)	
P2	0.7737 (2)	0.29796 (18)	0.71644 (18)	0.0231 (7)	
C1	0.7935 (9)	0.2299 (7)	1.0123 (7)	0.028 (3)	
O1	0.8519 (7)	0.1879 (6)	1.0251 (7)	0.059 (3)	
C2	0.7777 (9)	0.3741 (7)	1.0121 (7)	0.034 (3)	
O2	0.8262 (7)	0.4231 (5)	1.0217 (6)	0.043 (3)	
C3	0.9563 (9)	0.2289 (7)	0.8019 (9)	0.033 (3)	
O3	0.9828 (7)	0.1817 (5)	0.8526 (7)	0.050 (3)	
C4	0.9460 (8)	0.3751 (6)	0.8084 (7)	0.021 (3)	
O4	0.9641 (6)	0.4213 (5)	0.8581 (6)	0.036 (2)	
C11	0.6176 (9)	0.3548 (8)	1.0417 (8)	0.039 (4)	
H11	0.6117	0.4079	1.0406	0.046*	
C12	0.5664 (8)	0.3034 (8)	0.9779 (8)	0.037 (3)	
H12	0.5177	0.3162	0.9263	0.045*	
C13	0.5997 (9)	0.2295 (7)	1.0036 (8)	0.037 (4)	
H13	0.5791	0.1851	0.9710	0.044*	
C14	0.6680 (10)	0.2335 (8)	1.0851 (9)	0.043 (4)	
H14	0.6997	0.1919	1.1181	0.051*	
C15	0.6819 (9)	0.3087 (9)	1.1101 (8)	0.042 (4)	
H15	0.7253	0.3269	1.1622	0.051*	
C21	0.5872 (8)	0.3745 (6)	0.8036 (8)	0.025 (3)	
C22	0.5883 (9)	0.4404 (6)	0.8455 (8)	0.030 (3)	
H22	0.6269	0.4449	0.9037	0.036*	
C23	0.5331 (9)	0.5011 (7)	0.8036 (9)	0.040 (4)	
H23	0.5366	0.5462	0.8341	0.048*	
C24	0.4739 (9)	0.4976 (7)	0.7195 (8)	0.039 (4)	
H24	0.4359	0.5387	0.6924	0.047*	
C25	0.4724 (9)	0.4322 (7)	0.6769 (8)	0.037 (3)	
H25	0.4324	0.4283	0.6190	0.044*	
C26	0.5276 (8)	0.3711 (7)	0.7154 (7)	0.026 (3)	
H26	0.5259	0.3272	0.6835	0.031*	
C31	0.6003 (8)	0.2122 (6)	0.8099 (7)	0.021 (3)	
C32	0.5039 (8)	0.2071 (7)	0.7774 (7)	0.032 (3)	
H32	0.4698	0.2502	0.7787	0.038*	
C33	0.4592 (9)	0.1417 (6)	0.7443 (9)	0.036 (3)	
H33	0.3948	0.1411	0.7209	0.043*	
C34	0.5051 (9)	0.0776 (7)	0.7441 (11)	0.042 (4)	
H34	0.4725	0.0328	0.7214	0.051*	
C35	0.6013 (8)	0.0771 (7)	0.7775 (9)	0.032 (3)	
H35	0.6336	0.0325	0.7774	0.039*	
C36	0.6472 (9)	0.1438 (6)	0.8106 (8)	0.027 (3)	
H36	0.7116	0.1438	0.8343	0.032*	
C41	0.7122 (8)	0.3789 (6)	0.6556 (8)	0.026 (3)	
C42	0.7131 (9)	0.4462 (7)	0.6993 (9)	0.032 (3)	
H42	0.7455	0.4482	0.7590	0.038*	
C43	0.6678 (8)	0.5093 (7)	0.6568 (8)	0.032 (3)	
H43	0.6702	0.5541	0.6875	0.039*	
C44	0.6192 (10)	0.5081 (8)	0.5706 (8)	0.038 (4)	
H44	0.5868	0.5514	0.5423	0.045*	
C45	0.6178 (9)	0.4432 (8)	0.5254 (9)	0.038 (3)	
H45	0.5857	0.4418	0.4657	0.045*	
C46	0.6634 (8)	0.3818 (8)	0.5683 (8)	0.035 (3)	
H46	0.6618	0.3378	0.5365	0.042*	
C51	0.7135 (7)	0.2153 (6)	0.6561 (7)	0.023 (3)	
C52	0.6165 (8)	0.2150 (7)	0.6108 (8)	0.032 (3)	
H52	0.5823	0.2587	0.6099	0.038*	
C53	0.5731 (9)	0.1508 (7)	0.5685 (9)	0.037 (4)	
H53	0.5092	0.1515	0.5383	0.044*	
C54	0.6208 (10)	0.0849 (7)	0.5692 (8)	0.039 (4)	
H54	0.5901	0.0413	0.5402	0.046*	
C55	0.7130 (10)	0.0853 (7)	0.6129 (8)	0.037 (3)	
H55	0.7463	0.0409	0.6146	0.044*	
C56	0.7596 (9)	0.1499 (6)	0.6555 (7)	0.027 (3)	
H56	0.8237	0.1485	0.6841	0.032*	
C61	0.8766 (10)	0.3001 (10)	0.5953 (8)	0.051 (4)	
H61	0.8164	0.2895	0.5571	0.061*	
C62	0.9107 (10)	0.3710 (7)	0.6238 (8)	0.033 (3)	
H62	0.8780	0.4169	0.6098	0.040*	
C63	1.0042 (10)	0.3620 (9)	0.6785 (9)	0.046 (4)	
H63	1.0452	0.4014	0.7059	0.055*	
C64	1.0246 (10)	0.2864 (9)	0.6845 (8)	0.043 (4)	
H64	1.0818	0.2650	0.7180	0.051*	
C65	0.9461 (11)	0.2457 (9)	0.6322 (8)	0.044 (4)	
H65	0.9410	0.1929	0.6237	0.053*	
Fe3	0.72051 (13)	0.61469 (11)	0.12242 (12)	0.0352 (5)	
Cl31	0.6037 (3)	0.5782 (2)	0.0096 (2)	0.0469 (10)	
Cl32	0.6747 (3)	0.6715 (3)	0.2116 (3)	0.0857 (19)	
Cl33	0.7923 (3)	0.5112 (2)	0.1841 (3)	0.0557 (11)	
Cl34	0.8099 (3)	0.6898 (3)	0.0894 (3)	0.0657 (13)	
C5	0.8024 (11)	0.4708 (9)	0.3879 (11)	0.059 (5)	
H5	0.7751	0.4969	0.3320	0.071*	
Cl51	0.7504 (4)	0.3814 (3)	0.3768 (3)	0.0885 (17)	
Cl52	0.7830 (4)	0.5251 (3)	0.4605 (3)	0.0782 (15)	
Cl53	0.9181 (4)	0.4590 (4)	0.4120 (5)	0.119 (2)	

### Atomic displacement parameters (Å^2^)

**Table:**

	*U*^11^	*U*^22^	*U*^33^	*U*^12^	*U*^13^	*U*^23^
B1	0.020 (7)	0.038 (8)	0.024 (7)	−0.002 (7)	−0.003 (6)	−0.005 (7)
Fe1	0.0215 (9)	0.0301 (9)	0.0164 (9)	0.0028 (8)	0.0048 (7)	0.0030 (8)
Fe2	0.0220 (9)	0.0238 (9)	0.0221 (9)	−0.0005 (8)	0.0082 (7)	0.0007 (8)
P1	0.0193 (15)	0.0222 (15)	0.0151 (14)	0.0010 (14)	0.0040 (12)	0.0018 (13)
P2	0.0236 (16)	0.0216 (14)	0.0186 (15)	−0.0004 (15)	0.0024 (12)	0.0003 (14)
C1	0.038 (8)	0.036 (7)	0.013 (6)	−0.002 (6)	0.015 (6)	0.004 (5)
O1	0.055 (7)	0.068 (8)	0.054 (7)	0.048 (6)	0.023 (6)	0.034 (6)
C2	0.043 (9)	0.030 (7)	0.013 (6)	−0.004 (6)	−0.005 (6)	0.004 (5)
O2	0.051 (7)	0.046 (6)	0.026 (5)	−0.010 (5)	0.010 (5)	−0.002 (4)
C3	0.023 (7)	0.029 (7)	0.042 (8)	−0.013 (6)	0.008 (6)	−0.004 (6)
O3	0.045 (6)	0.033 (6)	0.063 (7)	0.017 (5)	0.011 (5)	0.022 (5)
C4	0.020 (6)	0.026 (6)	0.019 (6)	−0.001 (5)	0.009 (5)	0.005 (5)
O4	0.041 (6)	0.042 (5)	0.024 (5)	−0.010 (4)	0.011 (4)	−0.009 (4)
C11	0.026 (7)	0.051 (9)	0.032 (8)	−0.011 (6)	0.004 (6)	−0.016 (6)
C12	0.026 (7)	0.053 (8)	0.032 (7)	0.013 (7)	0.011 (6)	0.008 (7)
C13	0.047 (9)	0.049 (8)	0.012 (6)	−0.010 (7)	0.009 (6)	0.008 (5)
C14	0.038 (9)	0.058 (10)	0.031 (8)	0.000 (7)	0.014 (7)	0.027 (7)
C15	0.036 (8)	0.070 (11)	0.024 (7)	−0.009 (8)	0.015 (6)	0.013 (7)
C21	0.019 (6)	0.023 (6)	0.032 (7)	−0.008 (5)	0.010 (5)	0.006 (5)
C22	0.033 (8)	0.027 (6)	0.021 (7)	0.009 (6)	−0.001 (6)	0.000 (5)
C23	0.048 (9)	0.018 (6)	0.042 (9)	0.010 (6)	0.006 (7)	−0.001 (6)
C24	0.035 (8)	0.034 (7)	0.030 (8)	0.016 (6)	−0.007 (6)	0.008 (6)
C25	0.043 (9)	0.045 (8)	0.011 (6)	0.001 (6)	−0.001 (6)	0.004 (6)
C26	0.017 (6)	0.032 (7)	0.024 (7)	−0.016 (5)	0.004 (5)	−0.008 (5)
C31	0.030 (7)	0.021 (6)	0.012 (5)	0.007 (5)	0.006 (5)	−0.001 (5)
C32	0.035 (7)	0.031 (7)	0.035 (7)	−0.007 (6)	0.019 (6)	−0.007 (6)
C33	0.025 (7)	0.027 (7)	0.060 (10)	−0.004 (5)	0.023 (7)	−0.005 (6)
C34	0.032 (8)	0.018 (7)	0.078 (12)	−0.003 (6)	0.024 (8)	−0.004 (7)
C35	0.015 (7)	0.036 (7)	0.044 (8)	0.005 (5)	0.010 (6)	0.000 (6)
C36	0.031 (7)	0.023 (6)	0.028 (7)	0.011 (5)	0.013 (6)	0.006 (5)
C41	0.028 (7)	0.020 (6)	0.030 (7)	0.008 (5)	0.011 (6)	0.004 (5)
C42	0.022 (7)	0.035 (7)	0.035 (8)	0.005 (6)	0.008 (6)	0.013 (6)
C43	0.032 (8)	0.029 (7)	0.041 (8)	−0.014 (6)	0.019 (6)	0.007 (6)
C44	0.044 (9)	0.040 (8)	0.022 (7)	0.007 (7)	0.006 (6)	0.017 (6)
C45	0.034 (8)	0.045 (8)	0.030 (8)	0.017 (7)	0.008 (6)	0.005 (6)
C46	0.024 (7)	0.042 (8)	0.032 (7)	−0.002 (6)	0.004 (6)	−0.008 (6)
C51	0.019 (6)	0.021 (6)	0.022 (6)	−0.002 (5)	0.000 (5)	−0.003 (5)
C52	0.017 (6)	0.035 (7)	0.041 (8)	0.005 (5)	0.009 (6)	0.001 (6)
C53	0.023 (7)	0.032 (7)	0.048 (9)	−0.017 (6)	0.006 (6)	−0.028 (6)
C54	0.052 (10)	0.035 (8)	0.023 (7)	−0.025 (7)	0.008 (7)	−0.018 (6)
C55	0.046 (9)	0.023 (6)	0.031 (8)	−0.004 (6)	0.003 (6)	−0.010 (5)
C56	0.033 (7)	0.034 (7)	0.015 (6)	−0.002 (6)	0.011 (5)	−0.008 (5)
C61	0.051 (10)	0.079 (11)	0.022 (7)	0.006 (10)	0.014 (7)	−0.006 (9)
C62	0.053 (9)	0.020 (7)	0.033 (8)	−0.008 (6)	0.024 (7)	−0.002 (6)
C63	0.045 (9)	0.071 (11)	0.030 (8)	−0.023 (8)	0.023 (7)	−0.011 (7)
C64	0.040 (8)	0.066 (11)	0.026 (7)	0.020 (8)	0.018 (6)	0.001 (7)
C65	0.069 (11)	0.052 (9)	0.022 (8)	−0.015 (8)	0.029 (8)	−0.015 (7)
Fe3	0.0253 (10)	0.0465 (12)	0.0245 (10)	0.0111 (9)	−0.0003 (8)	−0.0047 (9)
Cl31	0.040 (2)	0.052 (2)	0.033 (2)	0.0042 (17)	−0.0024 (16)	−0.0157 (17)
Cl32	0.045 (3)	0.147 (5)	0.054 (3)	0.027 (3)	0.008 (2)	−0.050 (3)
Cl33	0.050 (2)	0.058 (2)	0.049 (2)	0.025 (2)	0.0098 (19)	0.0118 (19)
Cl34	0.051 (2)	0.075 (3)	0.055 (3)	−0.010 (2)	0.004 (2)	0.008 (2)
C5	0.055 (11)	0.063 (11)	0.045 (10)	0.007 (9)	0.003 (8)	0.012 (8)
Cl51	0.115 (4)	0.072 (3)	0.063 (3)	0.008 (3)	0.018 (3)	0.003 (3)
Cl52	0.105 (4)	0.061 (3)	0.076 (3)	0.016 (3)	0.044 (3)	0.014 (2)
Cl53	0.080 (4)	0.184 (7)	0.103 (5)	0.014 (4)	0.047 (4)	0.014 (5)

### Geometric parameters (Å, °)

**Table:**

B1—P2	1.967 (14)	C31—C36	1.430 (15)
B1—P1	1.967 (15)	C32—C33	1.370 (16)
B1—H1A	0.9900	C32—H32	0.9500
B1—H1B	0.9900	C33—C34	1.360 (17)
Fe1—C1	1.778 (14)	C33—H33	0.9500
Fe1—C2	1.793 (14)	C34—C35	1.420 (18)
Fe1—C13	2.079 (14)	C34—H34	0.9500
Fe1—C15	2.107 (13)	C35—C36	1.397 (17)
Fe1—C14	2.111 (13)	C35—H35	0.9500
Fe1—C12	2.111 (13)	C36—H36	0.9500
Fe1—C11	2.115 (15)	C41—C46	1.394 (17)
Fe1—P1	2.305 (3)	C41—C42	1.414 (17)
Fe2—C3	1.783 (14)	C42—C43	1.381 (16)
Fe2—C4	1.786 (12)	C42—H42	0.9500
Fe2—C62	2.102 (13)	C43—C44	1.375 (18)
Fe2—C64	2.104 (14)	C43—H43	0.9500
Fe2—C63	2.112 (15)	C44—C45	1.390 (19)
Fe2—C61	2.114 (13)	C44—H44	0.9500
Fe2—C65	2.128 (14)	C45—C46	1.361 (17)
Fe2—P2	2.301 (4)	C45—H45	0.9500
P1—C31	1.799 (12)	C46—H46	0.9500
P1—C21	1.848 (12)	C51—C56	1.383 (16)
P2—C41	1.825 (12)	C51—C52	1.441 (16)
P2—C51	1.841 (11)	C52—C53	1.387 (16)
C1—O1	1.153 (14)	C52—H52	0.9500
C2—O2	1.138 (14)	C53—C54	1.398 (18)
C3—O3	1.164 (14)	C53—H53	0.9500
C4—O4	1.140 (13)	C54—C55	1.370 (19)
C11—C12	1.416 (18)	C54—H54	0.9500
C11—C15	1.471 (18)	C55—C56	1.410 (15)
C11—H11	0.9500	C55—H55	0.9500
C12—C13	1.425 (18)	C56—H56	0.9500
C12—H12	0.9500	C61—C62	1.39 (2)
C13—C14	1.401 (18)	C61—C65	1.42 (2)
C13—H13	0.9500	C61—H61	0.9500
C14—C15	1.40 (2)	C62—C63	1.43 (2)
C14—H14	0.9500	C62—H62	0.9500
C15—H15	0.9500	C63—C64	1.38 (2)
C21—C22	1.374 (17)	C63—H63	0.9500
C21—C26	1.439 (16)	C64—C65	1.425 (19)
C22—C23	1.403 (16)	C64—H64	0.9500
C22—H22	0.9500	C65—H65	0.9500
C23—C24	1.383 (18)	Fe3—Cl32	2.193 (5)
C23—H23	0.9500	Fe3—Cl34	2.196 (5)
C24—C25	1.373 (18)	Fe3—Cl31	2.198 (4)
C24—H24	0.9500	Fe3—Cl33	2.211 (4)
C25—C26	1.394 (17)	C5—Cl52	1.703 (19)
C25—H25	0.9500	C5—Cl53	1.749 (18)
C26—H26	0.9500	C5—Cl51	1.775 (17)
C31—C32	1.426 (16)	C5—H5	1.0000
			
P2—B1—P1	123.2 (6)	C21—C22—C23	120.9 (11)
P2—B1—H1A	106.5	C21—C22—H22	119.5
P1—B1—H1A	106.5	C23—C22—H22	119.5
P2—B1—H1B	106.5	C24—C23—C22	122.5 (12)
P1—B1—H1B	106.5	C24—C23—H23	118.8
H1A—B1—H1B	106.5	C22—C23—H23	118.8
C1—Fe1—C2	92.6 (6)	C25—C24—C23	117.1 (11)
C1—Fe1—C13	104.6 (5)	C25—C24—H24	121.4
C2—Fe1—C13	159.4 (6)	C23—C24—H24	121.4
C1—Fe1—C15	112.0 (5)	C24—C25—C26	122.4 (11)
C2—Fe1—C15	97.4 (6)	C24—C25—H25	118.8
C13—Fe1—C15	65.9 (5)	C26—C25—H25	118.8
C1—Fe1—C14	90.1 (6)	C25—C26—C21	120.1 (11)
C2—Fe1—C14	131.8 (6)	C25—C26—H26	120.0
C13—Fe1—C14	39.1 (5)	C21—C26—H26	120.0
C15—Fe1—C14	38.7 (6)	C32—C31—C36	115.8 (11)
C1—Fe1—C12	143.6 (6)	C32—C31—P1	124.8 (9)
C2—Fe1—C12	123.7 (6)	C36—C31—P1	119.3 (9)
C13—Fe1—C12	39.8 (5)	C33—C32—C31	121.8 (12)
C15—Fe1—C12	66.1 (5)	C33—C32—H32	119.1
C14—Fe1—C12	65.7 (5)	C31—C32—H32	119.1
C1—Fe1—C11	152.7 (5)	C34—C33—C32	121.3 (13)
C2—Fe1—C11	92.6 (6)	C34—C33—H33	119.3
C13—Fe1—C11	67.0 (5)	C32—C33—H33	119.3
C15—Fe1—C11	40.8 (5)	C33—C34—C35	120.6 (12)
C14—Fe1—C11	66.8 (6)	C33—C34—H34	119.7
C12—Fe1—C11	39.2 (5)	C35—C34—H34	119.7
C1—Fe1—P1	91.0 (4)	C36—C35—C34	118.3 (12)
C2—Fe1—P1	91.5 (4)	C36—C35—H35	120.9
C13—Fe1—P1	99.4 (4)	C34—C35—H35	120.9
C15—Fe1—P1	154.9 (4)	C35—C36—C31	122.1 (11)
C14—Fe1—P1	136.6 (4)	C35—C36—H36	119.0
C12—Fe1—P1	89.5 (4)	C31—C36—H36	119.0
C11—Fe1—P1	115.7 (4)	C46—C41—C42	115.1 (11)
C3—Fe2—C4	94.3 (5)	C46—C41—P2	126.4 (10)
C3—Fe2—C62	158.3 (6)	C42—C41—P2	118.5 (9)
C4—Fe2—C62	100.5 (5)	C43—C42—C41	121.1 (13)
C3—Fe2—C64	93.7 (6)	C43—C42—H42	119.4
C4—Fe2—C64	114.8 (6)	C41—C42—H42	119.4
C62—Fe2—C64	65.7 (5)	C44—C43—C42	121.0 (13)
C3—Fe2—C63	125.6 (6)	C44—C43—H43	119.5
C4—Fe2—C63	89.4 (5)	C42—C43—H43	119.5
C62—Fe2—C63	39.7 (5)	C43—C44—C45	119.5 (12)
C64—Fe2—C63	38.3 (6)	C43—C44—H44	120.2
C3—Fe2—C61	128.0 (7)	C45—C44—H44	120.2
C4—Fe2—C61	137.6 (6)	C46—C45—C44	118.6 (13)
C62—Fe2—C61	38.4 (6)	C46—C45—H45	120.7
C64—Fe2—C61	65.3 (6)	C44—C45—H45	120.7
C63—Fe2—C61	64.9 (6)	C45—C46—C41	124.6 (13)
C3—Fe2—C65	94.2 (6)	C45—C46—H46	117.7
C4—Fe2—C65	153.3 (6)	C41—C46—H46	117.7
C62—Fe2—C65	65.7 (5)	C56—C51—C52	117.4 (10)
C64—Fe2—C65	39.3 (5)	C56—C51—P2	121.1 (9)
C63—Fe2—C65	65.2 (6)	C52—C51—P2	121.5 (9)
C61—Fe2—C65	39.2 (6)	C53—C52—C51	119.9 (11)
C3—Fe2—P2	92.8 (4)	C53—C52—H52	120.1
C4—Fe2—P2	90.7 (4)	C51—C52—H52	120.1
C62—Fe2—P2	102.7 (4)	C52—C53—C54	121.9 (11)
C64—Fe2—P2	153.0 (4)	C52—C53—H53	119.1
C63—Fe2—P2	141.5 (5)	C54—C53—H53	119.1
C61—Fe2—P2	90.3 (4)	C55—C54—C53	118.0 (11)
C65—Fe2—P2	114.1 (4)	C55—C54—H54	121.0
C31—P1—C21	105.3 (5)	C53—C54—H54	121.0
C31—P1—B1	110.1 (6)	C54—C55—C56	121.7 (12)
C21—P1—B1	112.4 (6)	C54—C55—H55	119.2
C31—P1—Fe1	108.6 (4)	C56—C55—H55	119.2
C21—P1—Fe1	111.2 (4)	C51—C56—C55	121.1 (12)
B1—P1—Fe1	109.0 (4)	C51—C56—H56	119.4
C41—P2—C51	105.4 (5)	C55—C56—H56	119.4
C41—P2—B1	111.4 (6)	C62—C61—C65	109.5 (13)
C51—P2—B1	111.2 (6)	C62—C61—Fe2	70.4 (8)
C41—P2—Fe2	108.3 (4)	C65—C61—Fe2	70.9 (8)
C51—P2—Fe2	112.6 (4)	C62—C61—H61	125.3
B1—P2—Fe2	107.9 (4)	C65—C61—H61	125.3
O1—C1—Fe1	176.9 (11)	Fe2—C61—H61	125.1
O2—C2—Fe1	178.0 (12)	C61—C62—C63	107.2 (13)
O3—C3—Fe2	176.5 (11)	C61—C62—Fe2	71.2 (8)
O4—C4—Fe2	177.2 (11)	C63—C62—Fe2	70.5 (8)
C12—C11—C15	105.5 (12)	C61—C62—H62	126.4
C12—C11—Fe1	70.3 (8)	C63—C62—H62	126.4
C15—C11—Fe1	69.3 (8)	Fe2—C62—H62	123.5
C12—C11—H11	127.2	C64—C63—C62	108.3 (13)
C15—C11—H11	127.2	C64—C63—Fe2	70.5 (9)
Fe1—C11—H11	124.8	C62—C63—Fe2	69.8 (8)
C11—C12—C13	109.0 (12)	C64—C63—H63	125.8
C11—C12—Fe1	70.6 (8)	C62—C63—H63	125.8
C13—C12—Fe1	68.9 (8)	Fe2—C63—H63	125.4
C11—C12—H12	125.5	C63—C64—C65	109.0 (13)
C13—C12—H12	125.5	C63—C64—Fe2	71.2 (8)
Fe1—C12—H12	126.6	C65—C64—Fe2	71.2 (8)
C14—C13—C12	108.2 (12)	C63—C64—H64	125.5
C14—C13—Fe1	71.7 (8)	C65—C64—H64	125.5
C12—C13—Fe1	71.4 (8)	Fe2—C64—H64	123.7
C14—C13—H13	125.9	C61—C65—C64	106.0 (13)
C12—C13—H13	125.9	C61—C65—Fe2	69.9 (8)
Fe1—C13—H13	122.7	C64—C65—Fe2	69.4 (7)
C15—C14—C13	108.8 (12)	C61—C65—H65	127.0
C15—C14—Fe1	70.5 (7)	C64—C65—H65	127.0
C13—C14—Fe1	69.2 (8)	Fe2—C65—H65	125.3
C15—C14—H14	125.6	Cl32—Fe3—Cl34	109.5 (2)
C13—C14—H14	125.6	Cl32—Fe3—Cl31	110.40 (17)
Fe1—C14—H14	126.3	Cl34—Fe3—Cl31	111.66 (18)
C14—C15—C11	108.3 (12)	Cl32—Fe3—Cl33	107.7 (2)
C14—C15—Fe1	70.8 (8)	Cl34—Fe3—Cl33	111.25 (19)
C11—C15—Fe1	69.9 (7)	Cl31—Fe3—Cl33	106.17 (17)
C14—C15—H15	125.9	Cl52—C5—Cl53	112.4 (10)
C11—C15—H15	125.9	Cl52—C5—Cl51	112.0 (10)
Fe1—C15—H15	125.0	Cl53—C5—Cl51	109.1 (9)
C22—C21—C26	117.0 (11)	Cl52—C5—H5	107.7
C22—C21—P1	122.4 (9)	Cl53—C5—H5	107.7
C26—C21—P1	120.5 (9)	Cl51—C5—H5	107.7
			
P2—B1—P1—C31	60.2 (11)	B1—P1—C21—C26	80.9 (11)
P2—B1—P1—C21	−57.0 (11)	Fe1—P1—C21—C26	−156.6 (8)
P2—B1—P1—Fe1	179.2 (8)	C26—C21—C22—C23	0.2 (19)
C1—Fe1—P1—C31	78.3 (6)	P1—C21—C22—C23	175.6 (11)
C2—Fe1—P1—C31	171.0 (6)	C21—C22—C23—C24	2(2)
C13—Fe1—P1—C31	−26.6 (6)	C22—C23—C24—C25	−2(2)
C15—Fe1—P1—C31	−78.1 (11)	C23—C24—C25—C26	0(2)
C14—Fe1—P1—C31	−12.9 (8)	C24—C25—C26—C21	2(2)
C12—Fe1—P1—C31	−65.3 (6)	C22—C21—C26—C25	−1.8 (18)
C11—Fe1—P1—C31	−95.5 (6)	P1—C21—C26—C25	−177.2 (10)
C1—Fe1—P1—C21	−166.2 (6)	C21—P1—C31—C32	−26.4 (11)
C2—Fe1—P1—C21	−73.5 (6)	B1—P1—C31—C32	−147.9 (10)
C13—Fe1—P1—C21	88.9 (6)	Fe1—P1—C31—C32	92.8 (10)
C15—Fe1—P1—C21	37.4 (11)	C21—P1—C31—C36	157.2 (10)
C14—Fe1—P1—C21	102.6 (7)	B1—P1—C31—C36	35.8 (11)
C12—Fe1—P1—C21	50.2 (6)	Fe1—P1—C31—C36	−83.5 (9)
C11—Fe1—P1—C21	20.0 (6)	C36—C31—C32—C33	−3.8 (17)
C1—Fe1—P1—B1	−41.6 (7)	P1—C31—C32—C33	179.8 (10)
C2—Fe1—P1—B1	51.0 (7)	C31—C32—C33—C34	3(2)
C13—Fe1—P1—B1	−146.6 (7)	C32—C33—C34—C35	−1(2)
C15—Fe1—P1—B1	161.9 (11)	C33—C34—C35—C36	0(2)
C14—Fe1—P1—B1	−132.9 (8)	C34—C35—C36—C31	−1(2)
C12—Fe1—P1—B1	174.7 (7)	C32—C31—C36—C35	3.1 (17)
C11—Fe1—P1—B1	144.5 (7)	P1—C31—C36—C35	179.8 (10)
P1—B1—P2—C41	59.4 (11)	C51—P2—C41—C46	−28.6 (13)
P1—B1—P2—C51	−57.8 (11)	B1—P2—C41—C46	−149.3 (12)
P1—B1—P2—Fe2	178.2 (8)	Fe2—P2—C41—C46	92.1 (12)
C3—Fe2—P2—C41	171.4 (6)	C51—P2—C41—C42	151.7 (11)
C4—Fe2—P2—C41	77.1 (6)	B1—P2—C41—C42	31.0 (12)
C62—Fe2—P2—C41	−23.9 (6)	Fe2—P2—C41—C42	−87.6 (10)
C64—Fe2—P2—C41	−84.8 (10)	C46—C41—C42—C43	0.4 (19)
C63—Fe2—P2—C41	−12.9 (8)	P2—C41—C42—C43	−179.9 (10)
C61—Fe2—P2—C41	−60.5 (7)	C41—C42—C43—C44	1(2)
C65—Fe2—P2—C41	−92.7 (6)	C42—C43—C44—C45	−2(2)
C3—Fe2—P2—C51	−72.5 (6)	C43—C44—C45—C46	1(2)
C4—Fe2—P2—C51	−166.8 (5)	C44—C45—C46—C41	0(2)
C62—Fe2—P2—C51	92.2 (5)	C42—C41—C46—C45	−1(2)
C64—Fe2—P2—C51	31.4 (10)	P2—C41—C46—C45	179.5 (11)
C63—Fe2—P2—C51	103.2 (8)	C41—P2—C51—C56	147.2 (11)
C61—Fe2—P2—C51	55.6 (7)	B1—P2—C51—C56	−92.0 (11)
C65—Fe2—P2—C51	23.4 (6)	Fe2—P2—C51—C56	29.3 (11)
C3—Fe2—P2—B1	50.6 (7)	C41—P2—C51—C52	−35.5 (11)
C4—Fe2—P2—B1	−43.7 (6)	B1—P2—C51—C52	85.3 (11)
C62—Fe2—P2—B1	−144.6 (6)	Fe2—P2—C51—C52	−153.4 (9)
C64—Fe2—P2—B1	154.5 (10)	C56—C51—C52—C53	−0.2 (19)
C63—Fe2—P2—B1	−133.7 (8)	P2—C51—C52—C53	−177.7 (11)
C61—Fe2—P2—B1	178.7 (7)	C51—C52—C53—C54	1(2)
C65—Fe2—P2—B1	146.5 (7)	C52—C53—C54—C55	0(2)
C1—Fe1—C11—C12	−113.7 (12)	C53—C54—C55—C56	−1(2)
C2—Fe1—C11—C12	145.6 (8)	C52—C51—C56—C55	−0.8 (18)
C13—Fe1—C11—C12	−36.8 (8)	P2—C51—C56—C55	176.6 (10)
C15—Fe1—C11—C12	−116.1 (12)	C54—C55—C56—C51	1(2)
C14—Fe1—C11—C12	−79.4 (9)	C3—Fe2—C61—C62	−155.8 (9)
P1—Fe1—C11—C12	52.7 (9)	C4—Fe2—C61—C62	19.0 (13)
C1—Fe1—C11—C15	2.4 (15)	C64—Fe2—C61—C62	−81.4 (9)
C2—Fe1—C11—C15	−98.3 (8)	C63—Fe2—C61—C62	−39.0 (9)
C13—Fe1—C11—C15	79.2 (8)	C65—Fe2—C61—C62	−119.9 (13)
C14—Fe1—C11—C15	36.6 (8)	P2—Fe2—C61—C62	110.4 (8)
C12—Fe1—C11—C15	116.1 (12)	C3—Fe2—C61—C65	−35.9 (12)
P1—Fe1—C11—C15	168.8 (7)	C4—Fe2—C61—C65	138.9 (10)
C15—C11—C12—C13	−2.4 (15)	C62—Fe2—C61—C65	119.9 (13)
Fe1—C11—C12—C13	58.3 (9)	C64—Fe2—C61—C65	38.5 (9)
C15—C11—C12—Fe1	−60.7 (9)	C63—Fe2—C61—C65	80.9 (10)
C1—Fe1—C12—C11	134.9 (10)	P2—Fe2—C61—C65	−129.7 (9)
C2—Fe1—C12—C11	−42.7 (10)	C65—C61—C62—C63	1.4 (16)
C13—Fe1—C12—C11	120.4 (11)	Fe2—C61—C62—C63	61.7 (9)
C15—Fe1—C12—C11	39.9 (8)	C65—C61—C62—Fe2	−60.4 (10)
C14—Fe1—C12—C11	82.5 (9)	C3—Fe2—C62—C61	60.8 (17)
P1—Fe1—C12—C11	−134.2 (8)	C4—Fe2—C62—C61	−167.1 (9)
C1—Fe1—C12—C13	14.5 (12)	C64—Fe2—C62—C61	80.3 (9)
C2—Fe1—C12—C13	−163.2 (8)	C63—Fe2—C62—C61	116.8 (12)
C15—Fe1—C12—C13	−80.5 (8)	C65—Fe2—C62—C61	37.0 (9)
C14—Fe1—C12—C13	−37.9 (8)	P2—Fe2—C62—C61	−73.9 (9)
C11—Fe1—C12—C13	−120.4 (11)	C3—Fe2—C62—C63	−56.0 (17)
P1—Fe1—C12—C13	105.4 (7)	C4—Fe2—C62—C63	76.1 (9)
C11—C12—C13—C14	3.4 (16)	C64—Fe2—C62—C63	−36.5 (8)
Fe1—C12—C13—C14	62.7 (10)	C61—Fe2—C62—C63	−116.8 (12)
C11—C12—C13—Fe1	−59.3 (10)	C65—Fe2—C62—C63	−79.8 (9)
C1—Fe1—C13—C14	71.5 (10)	P2—Fe2—C62—C63	169.3 (8)
C2—Fe1—C13—C14	−74.1 (17)	C61—C62—C63—C64	−1.9 (16)
C15—Fe1—C13—C14	−36.4 (9)	Fe2—C62—C63—C64	60.3 (10)
C12—Fe1—C13—C14	−117.3 (12)	C61—C62—C63—Fe2	−62.2 (9)
C11—Fe1—C13—C14	−81.0 (9)	C3—Fe2—C63—C64	38.8 (11)
P1—Fe1—C13—C14	165.0 (8)	C4—Fe2—C63—C64	133.6 (9)
C1—Fe1—C13—C12	−171.2 (8)	C62—Fe2—C63—C64	−119.0 (12)
C2—Fe1—C13—C12	43.2 (17)	C61—Fe2—C63—C64	−81.3 (9)
C15—Fe1—C13—C12	81.0 (8)	C65—Fe2—C63—C64	−37.8 (8)
C14—Fe1—C13—C12	117.3 (12)	P2—Fe2—C63—C64	−135.9 (8)
C11—Fe1—C13—C12	36.3 (8)	C3—Fe2—C63—C62	157.8 (8)
P1—Fe1—C13—C12	−77.7 (7)	C4—Fe2—C63—C62	−107.3 (8)
C12—C13—C14—C15	−2.9 (17)	C64—Fe2—C63—C62	119.0 (12)
Fe1—C13—C14—C15	59.5 (10)	C61—Fe2—C63—C62	37.8 (8)
C12—C13—C14—Fe1	−62.4 (10)	C65—Fe2—C63—C62	81.2 (8)
C1—Fe1—C14—C15	126.5 (9)	P2—Fe2—C63—C62	−16.9 (12)
C2—Fe1—C14—C15	32.9 (12)	C62—C63—C64—C65	1.7 (16)
C13—Fe1—C14—C15	−120.1 (13)	Fe2—C63—C64—C65	61.5 (10)
C12—Fe1—C14—C15	−81.5 (9)	C62—C63—C64—Fe2	−59.8 (10)
C11—Fe1—C14—C15	−38.5 (8)	C3—Fe2—C64—C63	−149.3 (9)
P1—Fe1—C14—C15	−141.9 (7)	C4—Fe2—C64—C63	−52.9 (10)
C1—Fe1—C14—C13	−113.4 (9)	C62—Fe2—C64—C63	37.8 (8)
C2—Fe1—C14—C13	153.0 (9)	C61—Fe2—C64—C63	80.2 (10)
C15—Fe1—C14—C13	120.1 (13)	C65—Fe2—C64—C63	118.6 (13)
C12—Fe1—C14—C13	38.6 (9)	P2—Fe2—C64—C63	107.0 (11)
C11—Fe1—C14—C13	81.5 (9)	C3—Fe2—C64—C65	92.1 (9)
P1—Fe1—C14—C13	−21.9 (12)	C4—Fe2—C64—C65	−171.4 (8)
C13—C14—C15—C11	1.4 (16)	C62—Fe2—C64—C65	−80.8 (9)
Fe1—C14—C15—C11	60.1 (9)	C63—Fe2—C64—C65	−118.6 (13)
C13—C14—C15—Fe1	−58.7 (10)	C61—Fe2—C64—C65	−38.4 (9)
C12—C11—C15—C14	0.7 (15)	P2—Fe2—C64—C65	−11.5 (15)
Fe1—C11—C15—C14	−60.7 (10)	C62—C61—C65—C64	−0.3 (15)
C12—C11—C15—Fe1	61.4 (9)	Fe2—C61—C65—C64	−60.3 (9)
C1—Fe1—C15—C14	−60.1 (9)	C62—C61—C65—Fe2	60.0 (10)
C2—Fe1—C15—C14	−155.9 (8)	C63—C64—C65—C61	−0.9 (15)
C13—Fe1—C15—C14	36.7 (8)	Fe2—C64—C65—C61	60.6 (9)
C12—Fe1—C15—C14	80.4 (9)	C63—C64—C65—Fe2	−61.5 (10)
C11—Fe1—C15—C14	118.8 (11)	C3—Fe2—C65—C61	152.4 (10)
P1—Fe1—C15—C14	94.4 (12)	C4—Fe2—C65—C61	−99.4 (15)
C1—Fe1—C15—C11	−178.8 (7)	C62—Fe2—C65—C61	−36.2 (9)
C2—Fe1—C15—C11	85.3 (8)	C64—Fe2—C65—C61	−116.9 (13)
C13—Fe1—C15—C11	−82.1 (8)	C63—Fe2—C65—C61	−80.1 (10)
C14—Fe1—C15—C11	−118.8 (11)	P2—Fe2—C65—C61	57.4 (10)
C12—Fe1—C15—C11	−38.4 (7)	C3—Fe2—C65—C64	−90.7 (9)
P1—Fe1—C15—C11	−24.4 (14)	C4—Fe2—C65—C64	17.5 (17)
C31—P1—C21—C22	145.8 (11)	C62—Fe2—C65—C64	80.7 (9)
B1—P1—C21—C22	−94.3 (12)	C63—Fe2—C65—C64	36.8 (9)
Fe1—P1—C21—C22	28.2 (12)	C61—Fe2—C65—C64	116.9 (13)
C31—P1—C21—C26	−39.0 (10)	P2—Fe2—C65—C64	174.3 (7)

## References

[bb1] Blessing, R. H. (1995). *Acta Cryst.* A**51**, 33–38. 10.1107/s0108767394005726 7702794

[bb2] Kückmann, T. I., Dornhaus, F., Bolte, M., Lerner, H.-W., Holthausen, M. C. & Wagner, M. (2007). *Eur. J. Inorg. Chem.* pp. 1989–2003.

[bb3] Sheldrick, G. M. (2008). *Acta Cryst.* A**64**, 112–122. 10.1107/S0108767307043930 18156677

[bb4] Spek, A. L. (2003). *J. Appl. Cryst.* **36**, 7–13.

[bb5] Stoe & Cie (2001). *X-AREA* Stoe & Cie, Darmstadt, Germany.

